# Resource planning of Chinese commercial banking systems using two-stage inverse data envelopment analysis with undesirable outputs

**DOI:** 10.1371/journal.pone.0218214

**Published:** 2019-06-13

**Authors:** Qingxian An, Xuyang Liu, Yongli Li, Beibei Xiong

**Affiliations:** 1 School of Business, Central South University, Changsha, P. R., China; 2 School of Management, Harbin Institute of Technology, Harbin, P. R., China; 3 School of Business Administration, Hunan University, Changsha, P. R., China; Shandong University of Science and Technology, CHINA

## Abstract

This paper develops two-stage inverse data envelopment analysis models with undesirable outputs to formulate resource plans for 16 Chinese listed commercial banks whose outputs are increased and overall efficiency is kept unchanged in the short term. We use these models to meet three different output targets, namely, increasing both the desirable and undesirable outputs by the same percentage, increasing these outputs by different percentages, and increasing only the desirable outputs while keeping the undesirable outputs unchanged. We find that operation cost and interest expense are more flexible than labor in the adjustment process and that deposits have no obvious law of change. The findings of this work provide some suggestions for bank managers.

## Introduction

Commercial banks play significant roles in an economy’s financial system, and their functions, service capabilities, and market status, are all rapidly evolving over time. Mainland China currently has 1 central bank, 3 policy banks, 6 large state-owned commercial banks (SOBs), 12 national joint-stock commercial banks (JSBs), approximately 137 city commercial banks, and approximately 224 rural commercial banks. The Industrial and Commercial Bank of China, the Agriculture Bank of China, the Bank of China, and the China Construction Bank are the four largest banks in the country and are all included in the list of 10 list largest banks in the world in 2012 in terms of market capitalization [1]. However, despite maintaining a good momentum in its development, the Chinese banking industry is also facing considerable pressures and risks. From the domestic perspective, JSBs with relatively small market shares have to compete with large-scale banks, while from the international perspective, since China’s accession to the World Trade Organization, foreign banks have also influenced some of the country’s domestic banks. Some risks can also be identified in the Chinese banking sector, including the large number of bad loans, the dominant position of loans to state-owned enterprises, and the influence of local government and Communist Party officials on these institutions’ lending decisions [2]. Therefore, measuring and improving the performance of the Chinese banking system have attracted the interest of many scholars [3,4]. However, under real conditions, banks are unable to improve their efficiency in the short term when attempting to fulfill temporary business tasks. Therefore, how these banks can formulate new resource (input) plans to accomplish their business goals in the short term is a topic worth exploring.

The performance of a system is usually evaluated via stochastic frontier analysis (SFA) and data envelopment analysis (DEA). SFA is a parametric methodology that considers the impact of random factors on output. However, this approach is only suitable for systems with only one output. Meanwhile, DEA is a data-driven approach that can measure the efficiency of those systems that include multiple inputs and outputs [5]. DEA has been used in many areas, including education [6], environment [7,8], manufacturing [9], transportation [10], and medical treatment [11,12]. The banking system is a complex system that uses multiple inputs to produce a few outputs, such as loans and interest income. Given the multi-parameter characteristics of the banking system, DEA shows superiority over SFA in measuring bank efficiency.

As a reverse application of DEA that is suitable for complex systems, inverse DEA was introduced in [13] to formulate resource plans or set short-term output goals for a decision making unit (DMU). Assuming that the efficiency of a DMU is known, inverse DEA can compute for the quantities of inputs when the outputs are set in advance by decisions makers (DMs) or make strategic outputs in advance when the resources are limited. In other words, under the efficiency-invariable principle, inverse DEA can help DMs make highly reasonable decisions in the short term. Many studies have applied inverse DEA [14–22] but ignored the internal structure of DMUs, which may deviate from the actual situation.

In this study, we examine how to formulate resource plans that can help those banks that adopt a two-stage system achieve their output targets in the short term while maintaining their overall efficiency score. To solve this problem, we build two-stage inverse DEA models that consider both the changes in desirable and undesirable outputs. This approach also addresses the gap in the literature that ignores network structure analysis in inverse DEA. When the output targets are predetermined, we use inverse DEA to formulate feasible plans for 16 Chinese commercial banking systems across three scenarios. The findings of this work also shed light on how banks or similar organizations can formulate temporary resource plans in the short term.

The rest of this paper is organized as follows. Section 2 reviews some existing research on evaluating the efficiency of banks and the development of inverse DEA method. Section 3 introduces the classical inverse DEA model and two-stage inverse models with undesirable outputs. Section 4 applies the proposed models in Chinese listed commercial banks. Section 5 concludes the paper.

## Literature review

In market-oriented reforms, banks play significant roles in the economy, and reasonably evaluating their performance is essential from the economic development perspective. Accordingly, many empirical studies have focused on evaluating the performance of banking systems by using the DEA introduced by Charnes et al. [5]. However, only few of these studies have formulated short-term resource plans or targets for these banks. To address this research gap, we integrate inverse DEA into a two-stage system to formulate resource plans or set targets for Chinese commercial banking systems.

The empirical research on Chinese banks can be classified into two categories, namely, efficiency changes and efficiency comparison. Studies under the efficiency changes category examine the changes in the efficiency of banks over a certain period and highlight the effects of significant events on such efficiency. For instance, after showing a steady increase in their efficiency during the early 1990s, Chinese banks showed a decreasing efficiency after the onset of major events, including the Asian financial crisis and the global economic slowdown [23]. These studies have also compared the efficiency of Chinese banks with different ownership types, especially SOBs and JSBs, and obtained contrasting findings. Some studies find that the SOBs are more efficient than other Chinese banks, while others find that JSBs outperform SOBs. Specifically, Chen et al. [23] and Yao et al. [24] found that most SOBs outperform JSBs in terms of efficiency, especially technical efficiency, whereas Ariff and Luc [25] concluded that JSBs have greater cost and profit efficiencies than SOBs between 1995 and 2004.

Whether it is to observe changes in bank efficiency or to compare the differences between bank efficiencies, it is necessary to first evaluate the relative efficiency of the banking system. With the in-depth analysis of the bank’s operating structure, more and more scholars regard the banking system as a two-stage structure. Wang et al. [1] measured the efficiency of the Chinese commercial banking system using two-stage DEA models. Boussemart et al. [26] decomposed banking performance based on the two-stage system. The first stage produces non-interest income and loans, and the second stage is the production of interest income from for loans. Izadikhah et al. [27] proposed a novel two-stage DEA production model based on two-stage network structure and apply it to banking industry. There are a lot of literatures on banking system using two-stage DEA [28–32].

By assuming that the known relative efficiency is constant, inverse DEA aims to formulate resource plans under an expanded output scale or to set output targets under reduced investments. Wei et al. [13] proposed systematic inverse DEA models under different efficiency situations. Hadi–Vencheh and Foroughi [33] extended the work of Wei et al. [13] to a more generalized case where some outputs are increasing while the other outputs are decreasing. Hadi–Vencheh et al. [20] also showed that the multiple objective optimization method proposed by Wei et al. [13] may fail in some situations. Zhang and Cui [34] recently extension and integration of inverse DEA in output- and input-oriented. Other studies have extended the application of inverse DEA to solving inverse problems that involve preference cone constraints [22], undesirable factors [21], sensitivity analysis [35], imprecise data [17], ambiguities [36], frontier changes [37], and available price information [38]. Hassanzadeh et al. [14] developed two inverse semi-oriented radial models for evaluating the sustainability of countries. In addition to its theoretical contributions, inverse DEA has also been applied in empirical research. For instance, Lin [39] used the inverse imprecise DEA to set revenue targets for the new stores of a home improvement company in Taiwan. Frija et al. [19] estimated the impact of irrigation pricing policies by using inverse DEA models. Gattoufi et al. [18] applied inverse DEA in studying the merger of banks. Amin et al. [40] developed the inverse GInvDEA model to illustrate a generalized restructuring. To the best of our knowledge, only three papers [17,21,41] have examined inverse DEA with undesired outputs.

Only few of those studies that examined inverse DEA have also applied inverse DEA in the field of banking. Gattoufi et al. [18] examined bank mergers and acquisitions by using inverse DEA. In their work, the efficiency of merged banks is predetermined by DMs, and the inverse DEA model formulates the resource plans or production targets according to this predetermined efficiency. Amin and Al-Muharrami [42] incorporated negative data into their study and developed an output-oriented inverse DEA model to set targets for merged banks. Their approach is based on the inverse DEA model of Gattoufi et al. [15] and the VRS SORM model proposed by Matin et al. [43]. Amin et al. [40] proposed the generalized inverse DEA, which is a generalized version of the model proposed by Gattoufi et al. [15]. Modhej et al. [44] combined inverse DEA with artificial neural networks to study the performance of 600 Iranian bank branches.

The above-mentioned inverse DEA research on banks have treated banking system as a black box and ignored its internal structure, which can lead to deviations in efficiency. Based on the biased efficiency values, the results obtained through inverse DEA may be out of line with the actual demand of banking system. In order to obtain the result value that is more realistic, we comprehensively refer to the existing research on bank efficiency evaluation[1,26–32]. We believe that the two-stage structure should be used to evaluate bank efficiency and efficiency result is used as the efficiency basis of inverse DEA. This research idea is different from the existing research on inverse DEA applied to banks. In our study, the banking system is treated as a two-stage system with profitability stage and marketability stage [45], instead of treating the banking system as a box. We can measure the performance of banking system We use a two-stage DEA to more accurately measure the banking system, and using inverse DEA to get a more realistic and effective resource planning.

In sum, previous studies on inverse DEA have treated DMUs as a black box, and no two-stage study on the development of inverse DEA has been published for now. Moreover, very few studies have presented a theoretical expansion and application of inverse DEA models with undesirable outputs.

## Methodology

### Classical inverse DEA

Assume that a set of DMU_*j*_ (*j* = 1,2, …, *n*) ignoring the internal structure only has *m* inputs *x*_*ij*_ (i = 1,2, …, *m*) and *s* desirable outputsn *y*_*rj*_ (r = 1,2, …, *s*). Then, the efficiency of a specific DMU, called DMU_0_, can be calculated by using the CCR model (1).

$$\begin{array}{l} \text{min}\theta_{0}\\ \text{s}.\text{t}.\\ \sum_{j=1}^{n}\lambda_{j}x_{ij}\leq \theta_{0}x_{i0},\;i=1,2,\ldots ,m\\ \sum_{j=1}^{n}\lambda_{j}y_{rj}\geq y_{r0},\;r=1,2,\ldots ,\text{s}\\ \lambda_{j}\geq 0,\;j=1,2,\ldots ,n \end{array}$$ (1)

where *λ*_*j*_ denotes the intensity vectors, and *θ*_0_ denotes the efficiency score of DMU_0_. Let $\theta_{0}^{CCR}$ represent the optimal efficiency value of DMU_0_ in model (1).

**Definition 1**. If the optimal value $\theta_{0}^{CCR}$ of model (1) is unity, then the DMU_0_ is considered (weakly) efficient.

Let DMU_0_ hold its efficiency $\theta_{0}^{CCR}$ and increase its outputs from *y* to *β*_*r0*_ = *y*_*r0*_ + Δ*y*_*r*0_. The DMU_0_ before and after the changes in outputs are denoted by initial DMU_0_ and perturbed DMU_0_, respectively. Wei et al. [13] proposed an inverse DEA model that minimizes the new resource plans (*α*_10_, *α*_20_, …, *α*_m0_) of perturbed DMU_0_, see model (2).

$$\begin{array}{l} \text{min}\left(\alpha_{10},\alpha_{20},\ldots ,\alpha_{m0}\right)\\ \text{s}.\text{t}.\\ \sum_{j=1}^{n}\lambda_{j}x_{ij}\leq \theta_{0}^{CCR}\alpha_{i0},\;i=1,2,\ldots ,m\\ \sum_{j=1}^{n}\lambda_{j}y_{rj}\geq \beta_{r0},\;r=1,2,\ldots ,s\\ \lambda_{j}\geq 0,\;j=1,2,\ldots ,n \end{array}$$ (2)

where the objective function is a multi-objective function that attempts to minimize the resources of DMU_0_. The two constraints ensure that the efficiency of the perturbed DMU_0_ is the same as that of the initial DMU_0_.

**Definition 2**. Suppose that ($\alpha_{i0}^{*},\lambda_{j}^{*}$) is a feasible solution model (2). If no feasible solution (${\bar{\alpha }}_{i0},{\bar{\lambda }}_{j}$) exists such that ${\bar{\alpha }}_{i0}<\alpha_{i0}^{*}$ for all *i* = 1,2, …*m* in model (2), then ($\alpha_{i0}^{*},\lambda_{j}^{*}$) is the weak Pareto optimal solution for model (2).

Model (2) is a multi-objective linear program (MOLP) that cannot easily obtain a Pareto optimal solution. To address this problem, Lertworasirikul et al. [46] assigned a set of positive weights to each objective to find a Pareto optimal solution. We apply this method and propose model (3) as follows to find the Pareto optimal solution of model (2).

$$\begin{array}{l} \text{min}W^{\text{T}}\left(\alpha_{10},\alpha_{20},\ldots ,\alpha_{m0}\right)\\ \text{s}.\text{t}.\\ \sum_{j=1}^{n}\lambda_{j}x_{ij}\leq \theta_{0}^{CCR}\alpha_{i0},\;i=1,2,\ldots ,m\\ \sum_{j=1}^{n}\lambda_{j}y_{rj}\geq \beta_{r0},\;r=1,2,\ldots ,s\\ \lambda_{j}\geq 0,\;j=1,2,\ldots ,n \end{array}$$ (3)

where *W*^*T*^ = *w*_*1*_, *w*_*2*_, …, *w*_*m*_ denotes the relative importance of each input (*α*_10_, *α*_20_, …, *α*_m0_), respectively. The value of these weights is artificially set.

### Two-stage inverse DEA with undesirable outputs

**Fig 1:**
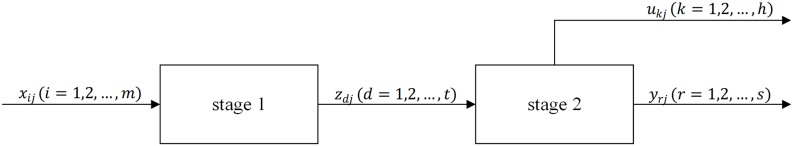
A two-stage system with undesirable outputs.

Suppose that *n* DMUs need to be evaluated as shown in Fig 1. For each DMU_*j*_ (j = 1,2 …, *n*), stage 1 consumes *m* inputs, *x*_*ij*_ (*i* = 1,2, …, *m*), and produces *t* outputs, *z*_*dj*_ (*d* = 1,2, …, *t*), which are called intermediate measures. Afterward, these *t* intermediate measures are treated as inputs in stage 2, which produces *s* desirable outputs, *y*_*rj*_ (*r* = 1,2, …, *s*), and *h* undesirable outputs, *u*_*kj*_ (*k* = 1,2, …, *h*).

Existing inverse DEA studies are based on envelope models, and require the original efficiency level of the DMU as a known parameter. Chen et al. [47] proposed the following input-oriented network-DEA model to measure the performance of the two-stage system without undesirable outputs. Their model is envelopment-based and can measure the overall efficiency of two-stage system. Therefore, we choose model (4) as the basis to measure the overall performance of banking system.

$$\begin{array}{l} \text{min}\theta_{0}\\ \text{s}.\text{t}.\\ \sum_{j=1}^{n}\lambda_{j}x_{ij}\leq \theta_{0}x_{i0},\;i=1,2,\ldots ,m\\ \sum_{j=1}^{n}\lambda_{j}z_{dj}\geq {\tilde{z}}_{do},\;d=1,2,\ldots ,t\\ \sum_{j=1}^{n}\pi_{j}z_{dj}\leq {\tilde{z}}_{do},\;d=1,2,\ldots ,t\\ \sum_{j=1}^{n}\pi_{j}y_{rj}\geq y_{r0},\;r=1,2,\ldots ,s\\ \lambda_{j}\geq 0,\;j=1,2,\ldots ,n\\ \pi_{j}\geq 0,\;j=1,2,\ldots ,n\\ {\tilde{z}}_{do}\geq 0,\;d=1,2,\ldots ,t \end{array}$$ (4)

where *λj* (*j* = 1,2, …, *n*) and *πj* (*j* = 1,2, …, *n*) denote the intensity vectors corresponding to stages 1 and 2, while ${\tilde{z}}_{d0}\left(d\;=\;1,2,\ldots ,t\right)$ denotes the set of new intermediate measures to be determined.

The undesirable outputs of banks have a weak disposability according to Liu et al. [48]. A weak disposability means that all inputs or outputs must be increased or decreased by the same percentage. For example, a power plant burns coal to produce electricity (desirable output) and emit sulfur dioxide (undesirable output). The outputs of this power plant have weak disposability, which implies that if exhaust gas is expected to be reduced by a certain percentage, then the electricity must be reduced by the same percentage. For the weak disposability of outputs, we assume that if (*X*, *Y*) ∈ *P* and 0 < *δ* < 1, then (*X*, *δY*) ∈ *P*, where *P* is the production possibility set and *δ* is the change ratio (Liu et al., 2015). To estimate the efficiency of the two-stage system with undesirable outputs that satisfies the weakly free disposability shown in Fig 1, we extend model (4) by adding another constraint for undesirable outputs. Färe and Grosskopf [49] modelled the following strict equality constriction to limit the undesirable outputs with weak disposability.

$$\begin{array}{l} \text{min}\theta_{0}\\ \text{s}.\text{t}.\\ \sum_{j=1}^{n}\lambda_{j}x_{ij}\leq \theta_{0}x_{i0},\;i=1,2,\ldots ,m\\ \sum_{j=1}^{n}\lambda_{j}z_{dj}\geq {\tilde{z}}_{do},\;d=1,2,\ldots ,t\\ \sum_{j=1}^{n}\pi_{j}z_{dj}\leq {\tilde{z}}_{do},\;d=1,2,\ldots ,t\\ \sum_{j=1}^{n}\pi_{j}y_{rj}\geq y_{r0},\;r=1,2,\ldots ,s\\ \sum_{j=1}^{n}\pi_{j}u_{kj}=u_{k0},\;k=1,2,\ldots ,h\\ \lambda_{j}\geq 0,\;j=1,2,\ldots ,n\\ \pi_{j}\geq 0,\;j=1,2,\ldots ,n\\ {\tilde{z}}_{do}\geq 0,\;d=1,2,\ldots ,t \end{array}$$ (5)

which optimal $\theta_{0}^{*}$ represents the overall efficiency score of the two-stage system with undesirable outputs.

**Definition 3.** If the optimal value $\theta_{0}^{*}$ of model (5) is unity, then DMU_0_ is (weakly) overall efficient.

If the two-stage system DMU_0_ increases its undesirable outputs from *u*_*k0*_ to *μ*_*k*0_ = *u*_*k*0_ + Δ*u*_*k*0_ and increases its desirable outputs from *y*_*r0*_ to *β*_*r*0_ = *y*_*r*0_ + Δ*y*_*r*0_ without changing its efficiency score $\theta_{0}^{*}$, then how many new inputs and intermediate measures will be produced for DMU_0_? Ghiyasi [41] proposed the following model (6) for formulating new input plans without considering the internal structure of DMU_0_.

$$\begin{array}{l} \text{min}\left(\alpha_{10},\alpha_{20},\ldots ,\alpha_{m0}\right)\\ \text{s}.\text{t}.\\ \sum_{j=1}^{n}\lambda_{j}x_{ij}\leq \theta_{0}^{*}\alpha_{i0},\;i=1,2,\ldots ,m\\ \sum_{j=1}^{n}\lambda_{j}y_{rj}\geq \beta_{r0},\;r=1,2,\ldots ,s\\ \sum_{j=1}^{n}\lambda_{j}u_{kj}=\mu_{k0},\;k=1,2,\ldots ,h\\ \lambda_{j}\geq 0,\;j=1,2,\ldots ,n \end{array}$$ (6)

Suppose that (*γ*_10_, *γ*_20_, …, *γ*_*t*0_) denotes the new intermediate measures of perturbed DMU_0_. We extend model (6) to the following two-stage inverse DEA model (7) with undesirable outputs.

$$\begin{array}{l} \text{min}\left(\alpha_{10},\alpha_{20},\ldots ,\alpha_{m0}\right)\\ \text{s}.\text{t}.\\ \sum_{j=1}^{n}\lambda_{j}x_{ij}\leq \theta_{0}^{*}\alpha_{i0},\;i=1,2,\ldots ,m\\ \sum_{j=1}^{n}\lambda_{j}z_{dj}\geq \gamma_{d0},\;d=1,2,\ldots ,t\\ \sum_{j=1}^{n}\pi_{j}z_{dj}\leq \gamma_{d0},\;d=1,2,\ldots ,t\\ \sum_{j=1}^{n}\pi_{j}y_{rj}\geq \beta_{r0},\;r=1,2,\ldots ,s\\ \sum_{j=1}^{n}\pi_{j}u_{kj}=\mu_{k0},\;k=1,2,\ldots ,h\\ \lambda_{j}\geq 0,\;j=1,2,\ldots ,n\\ \pi_{j}\geq 0,\;j=1,2,\ldots ,n\\ \gamma_{d0}\geq 0,\;d=1,2,\ldots ,t \end{array}$$ (7)

Given that model (7) is a MOLP whose Pareto optimal solution cannot be easily found, similar to model (3), we give a set weight *W*^*T*^ = *w*_1_, *w*_2_, …, *w*m to each objective function to find a unique solution.

**Definition 4.** Suppose that ($\alpha_{i0}^{*},\lambda_{j}^{*},\pi_{j}^{*},\gamma_{d0}^{*}$) is a feasible solution model (7) that uses weight *W*^*T*^ = *w*_1_, *w*_2_, …, *w*m. If there is no feasible solution (${\bar{\alpha }}_{i0},{\bar{\lambda }}_{j},{\bar{\pi }}_{j},{\bar{\gamma }}_{d0}$) such that ${\bar{\alpha }}_{i0}<\alpha_{i0}^{*}$ for *i* = 1,2, …, m in model (7)), then ($\alpha_{i0}^{*},\lambda_{j}^{*},\pi_{j}^{*},\gamma_{d0}^{*}$) is a weak Pareto optimal solution for model (7).

We then check whether the efficiency of the perturbed DMU_0_ is consistent with that of the initial DMU_0_. Suppose that DMU_*n*+1_ represents the perturbed DMU_0_. The efficiency of DMU_*n*+1_ can be estimated by the following model.

$$\begin{array}{l} \text{min}\theta_{0}\\ \text{s}.\text{t}.\\ \sum_{j=1}^{n}\lambda_{j}x_{ij}+\lambda_{n+1}\alpha_{i0}\leq \theta_{0}\alpha_{i0},\;i=1,2,\ldots ,m\\ \sum_{j=1}^{n}\lambda_{j}z_{dj}+\lambda_{n+1}\gamma_{d0}\geq {\tilde{\gamma }}_{d0},\;d=1,2,\ldots ,t\\ \sum_{j=1}^{n}\pi_{j}z_{dj}+\pi_{n+1}\gamma_{d0}\leq {\tilde{\gamma }}_{d0},\;d=1,2,\ldots ,t\\ \sum_{j=1}^{n}\pi_{j}y_{rj}+\pi_{n+1}\beta_{r0}\geq \beta_{r0},\;r=1,2,\ldots ,s\\ \sum_{j=1}^{n}\pi_{j}u_{kj}+\pi_{n+1}\mu_{k0}=\mu_{k0},\;k=1,2,\ldots ,h\\ \lambda_{j}\geq 0,\;j=1,2,\ldots ,n\text{+}1\\ \pi_{j}\geq 0,\;j=1,2,\ldots ,n\text{+1} \end{array}$$ (8)

**Theorem 1.** Suppose that $\theta_{0}^{*}$ is the optimal efficiency of model (5) for DMU_0_, that the desirable outputs increase from *y*_*r*0_ to *β*_*r*0_ = *y*_*r*0_ + Δ*y*_*r*0_ (Δ*y*_*r*0_ ≥ 0 and Δ*y*_*r*0_ ≠ 0), and that the desirable outputs increase from u_*k*0_ to *μ*_*k*0_ = *u*_*k*0_ + Δ*u*_*k*0_ (Δ*u*_*k*0_ ≥ 0 and Δ*u*_*k*0_ ≠ 0). If ($\alpha_{i0}^{*},\lambda_{j}^{*},\pi_{j}^{*},\gamma_{d0}^{*}$) is a weak Pareto optimal solution of the MOLP model (7), then the optimal value of model (8) is also $\theta_{0}^{*}$.

**Proof:** See Appendix.

## Application in Chinese listed commercial banks

### Input and output selection

The classification of inputs and outputs for evaluating the performance of banks have been extensively discussed in the literature. Several methodologies have also produced different classifications of inputs and outputs. Two commonly accepted methods, namely, the intermediation approach and production approach, have been used to evaluate the efficiency of banks. The main difference between these methods lies in their categories of deposits [50]. Specifically, the intermediation approach considers banks as financial intermediaries that gather deposits as inputs for making loans and investments (outputs), whereas the production approach assumes that deposits and loans are outputs produced by labor and capital. Although these methods examine the banking system from different perspectives, they generally view the bank as a black box and ignore its internal structure. Many studies on the banking system have conducted their research in two stages [51–54]. Stage 1 applies the production approach, which produces deposits, whereas stage 2 applies the intermediation approach, which uses deposits to create value.

When selecting other input and output indicators, previous studies have adopted very similar sets of inputs and outputs, although these indicators may show some dissimilarities when examined in detail. Therefore, in the two-stage banking system, cost or capital is usually treated as an input similar to labor, while the output can be divided into desirable and undesirable outputs. Desirable outputs mainly include interest and non-interest income, while undesirable outputs include non-performing (bad) loans. These indicators are significant items and are commonly used as variables in evaluating the performance of a banking system. Table 1 presents the variables used in this work in detail.

**Table 1:** Variables selected for this study.

	Variables	Descriptions
Inputs	Operation cost (*x*_1_)	Consumption by banks
	Interest expense (*x*_2_)	Expenses for all deposit portfolios
	Labor (*x*_3_)	Number of full-time staffs
Intermediate measure	Deposits (*z*_1_)	Bank deposits at the end of the year
Outputs	Interest income (*y*_1_)	Interest earned through loans, etc.
	Non-interest income (*y*_2_)	Other income excluding interest
	Non-performing loan balance (*u*_1_)	Non-performing loans at the end of the year

Notes: Labor is expressed in persons, while the other parameters are expressed in 100 million RMB.

### Data and sample

This section analyzes the data for 16 Chinese listed commercial banks, including 4 commercial SOBs, 9 commercial JSBs, and 3 city commercial banks. The 2013 data for these banks is collected from the Listed Commercial Bank of China Financial Reporting Database of the China Merchants Bank. The detailed data for inputs, intermediate measures, and outputs are presented in Table 2. The names and DMU numbers of these banks are listed in Table 3.

**Table 2:** The data for the 16 Chinese listed commercial banks in 2013.

DMU	*x*_*1*_	*x*_*2*_	*x*_*3*_	*z*_*1*_	*y*_*1*_	*y*_*2*_	*u*_*1*_
1	1768.29	3237.76	441902	146208.3	7671.11	1477.93	191.14
2	1693.97	2371.82	473766	118114.1	6133.84	875.85	19.33
3	1478.42	2354.1	251617	100977.9	5189.95	1245.09	78.23
4	1557.79	2567.09	368410	122230.4	6462.53	1208.98	106.46
5	538.12	1286.34	99919	41578.33	2592.92	341.7	73.15
6	458.96	745.82	51667	27752.76	1734.95	342.05	66.38
7	328.45	776.47	38803	26516.78	1633.35	191.34	77.11
8	266.05	926.27	38976	24196.96	1778.04	151.64	41.39
9	291.9	1037.57	33134	21703.45	1896.02	236.25	50.45
10	380.9	991.21	53064	21466.89	1821.54	332.01	28.81
11	212.79	524.14	28369	12170.02	931.02	115.86	6.75
12	176.23	373.51	25043	11775.92	762.53	63.62	11.04
13	207.81	692.2	31464	16052.78	1200.82	145.8	24.16
14	78.41	315.96	9193	8344.8	578.81	44.32	8.44
15	32.55	116.72	4357	2601.49	207.68	14.29	2.64
16	44.50	122.36	6310	2339.38	234.95	14.16	4.17

**Table 3:** Names and DMU numbers of the selected banks.

DMU	Bank	DMU	Bank
1	Industrial and Commercial Bank of China	9	Industrial Bank Co,. Ltd
2	Agricultural Bank of China	10	China Minsheng Banking
3	Bank of China	11	Ping An Bank
4	China Construction Bank	12	Huaxia Bank
5	Bank of Communications	13	China Everbright Bank
6	China Merchants Bank	14	Bank of Beijing
7	China CITIC Bank	15	Bank of Nanjing
8	Shanghai Pudong Development Bank	16	Bank of Ningbo

### Efficiency comparison

We initially evaluate the performance of the 16 Chinese listed commercial banks, whose data are presented in Table 4, by using the black box model and two-stage model. We apply each of these models in cases with and without undesirable outputs. Table 3 presents the efficiencies of these 16 banks. First, the number of efficient DMUs identified by the black box model in cases without and with undesirable outputs are 10 and 12, respectively, which are far greater than the number obtained by the two-stage model (0 and 1 for these two cases, respectively). This finding indicates that the discernment of the black box model is not as good as that of the two-stage model. Therefore, adopting the two-stage model to evaluate the efficiency of DMUs can obtain more authentic results that help the inverse DEA model produce highly reasonable recommendations. Second, the efficiency scores of DMUs as obtained by the two-stage model may considerably vary between these two cases. For example, the efficiency of DMU 3 is 0.797 in the case without undesirable outputs, but DMU 2 emerges as the only efficient DMU in the case with undesirable outputs. Similarly, the efficiencies of DMUs 2, 4, 5, 6, and 7 substantially increases in the latter stage. In other words, although the same model (the two-stage model) is used in the calculation, the efficiencies significantly vary in the two cases. In sum, the two-stage model is more effective than the black box model in terms of its recognition ability, and the undesirable outputs can greatly influence the evaluation results. In this case, when formulating an efficiency rule that needs to be followed by inverse DEA models, we must choose a highly realistic situation for evaluating the efficiency of a DMU (i.e., the two-stage model with undesirable outputs).

**Table 4:** Comparison of the black box model and two-stage model in terms of efficiency.

DMU	Black box model efficiency		Two-stage model overall efficiency	
	without undesirable outputs	with undesirable outputs	without undesirable outputs	with undesirable outputs
1	1.000	1.000	0.654	0.868
2	1.000	1.000	0.581	0.901
3	1.000	1.000	0.797	1.000
4	1.000	1.000	0.640	0.916
5	0.937	1.000	0.615	0.895
6	1.000	1.000	0.797	0.946
7	1.000	1.000	0.666	0.932
8	1.000	1.000	0.689	0.726
9	1.000	1.000	0.727	0.762
10	1.000	1.000	0.655	0.655
11	0.854	0.872	0.573	0.621
12	0.931	0.939	0.573	0.718
13	0.921	0.986	0.639	0.707
14	1.000	1.000	0.708	0.709
15	0.939	0.956	0.638	0.648
16	0.944	1.000	0.594	0.594

### Resource planning of Chinese commercial banks

DMs may set three types of output targets, namely, increasing both desirable and undesirable outputs by the same percentage, increasing both outputs by different percentages, and increasing the desirable outputs while keeping the undesirable outputs unchanged. The first target is observed in the situation where the DMs are not very concerned about the increasing ratio of undesirable outputs (non-performing loan balance). This target is acceptable as long as the ratio of undesirable outputs does not exceed that of desirable outputs (e.g., both the desirable and undesirable outputs increase by 15%). The second target is observed when DMs expect the undesirable outputs to increase by a lower percentage compared with the desirable outputs (e.g., the desirable outputs increase by 15%, while the undesirable outputs only increase by 5%). In the third target, only the desirable outputs are allowed to increase. The first and third targets represent an extreme situation. In real-world situations, DMs always hope that the undesired output will increase as little as possible; therefore, they do not favor a same percentage increase in both desirable and undesirable outputs. The third target is also unrealistic. Therefore, we only discuss the second targets in this paper, i.e. increasing both outputs with the percentage increase of desirable outputs higher than that of undesirable outputs.

We increase the desirable and undesirable outputs by different percentages, with the percentage increase of desirable outputs higher than that of undesirable outputs. This target is more favored by DMs given that people always expect to increase the desirable outputs as much as possible while minimizing the increase in the undesirable outputs. We therefore increase the desirable outputs by 15% and the undesirable outputs by 10%, 5% and 1%, respectively.

#### Scenario 1

We increase desirable outputs by 15% and undesirable outputs by 10% and analyze the new resource plans. We assume that the weight of each input is 1. Table 5 reports the new number of inputs and the net percentage increase. First, from the weak disposal perspective, setting new output targets is not possible for some DMUs, such as DMUs 9 and 10. A similar situation is observed in [41]. Specifically, when Ghiyasi [41] expanded both the desirable and undesirable outputs with different ratios, P17 and P12 (the two DMUs in [41]) were unable to meet the output expansion plan because they are both efficient DMUs without input waste in their input-oriented models. Meanwhile, in our work, DMUs 9 and 10 are inefficient DMUs for two-stage models yet are efficient DMUs for black box models. No feasible solution (NF) is obtained because the constraints for the undesirable outputs are used as very strict equality constraints in model (6). Therefore, no feasible solution may satisfy the equality constraint specified in model (6). The following analysis does not include DMU 9 and DMU 10.

**Table 5:** Plans of inputs and intermediate measure for increasing the desirable outputs by 15% and undesirable outputs by 10%.

DMU	Plans of inputs and intermediate measure				Net percentage increase (%)			
	α_1_	α_2_	α_3_	γ	P_1_	P_2_	P_3_	P_4_
1	2067.52	3691.79	441902.00	141643.66	16.92	14.02	0.00	-3.12
2	1693.97	2739.11	473766.00	118092.03	0.00	15.49	0.00	-0.02
3	1478.42	2828.94	251617.00	112863.95	0.00	20.17	0.00	11.77
4	1726.63	3086.91	368410.00	124812.59	10.84	20.25	0.00	2.11
5	648.49	1392.05	99919.00	46436.90	20.51	8.22	0.00	11.69
6	458.96	870.17	51667.00	29326.91	0.00	16.67	0.00	5.67
7	329.33	971.27	38803.00	27541.56	0.27	25.09	0.00	3.86
8	330.87	991.26	38976.00	21728.34	24.37	7.02	0.00	-10.20
9	NF	NF	NF	NF	NF	NF	NF	NF
10	NF	NF	NF	NF	NF	NF	NF	NF
11	240.29	602.42	28369.00	12378.15	12.92	14.94	0.00	1.71
12	190.08	433.70	25043.00	10980.98	7.86	16.11	0.00	-6.75
13	266.74	719.85	31464.00	16193.93	28.36	4.00	0.00	0.88
14	91.06	366.90	10675.13	6871.31	16.12	16.12	16.12	-17.66
15	37.15	147.45	4357.00	2537.57	14.13	26.32	0.00	-2.46
16	53.53	152.00	6310.00	2799.23	20.28	24.22	0.00	19.66

Note: NF means No feasible solution.

Second, the third input (labor) of most DMUs does not change, except for DMUs 14. This situation is rational to some extent. Inverse DEA has a short-run time condition, and a bank cannot easily increase its number of full-time employees temporarily given that recruiting new employees is a complex and lengthy process, especially for commercial SOBs. Conversely, banks can easily change their operation (*x*_1_) and interest costs (*x*_2_). If DMs still want to change the number of their staff, then they can do so by changing the dimension of parameters, such as in units of hundreds of people.

Third, with the exception of DMU 11, the net percentage increase of at least one input for most DMUs is no less than 15%. In other words, when we increase desirable outputs by 15% and undesirable outputs by 10% and keep the efficiency score unchanged, the net percentage increase in inputs usually exceed 15%. Specially, the inputs of DMU 14 increase by 16.12%, which is relatively balanced. There are 8 DMUs’ certain input needs to increase by more than 20%. Among these DMUs, the first input (operation cost) of DMU 13 increases by 28.36%, which is the highest recorded percentage. When changing the variables individually, DMUs 13 and 7 face greater pressure in increasing their operation costs and interest expenses, respectively. Specifically, DMU 13 has to increase its operation cost to 128.36% of its original value (i.e., 266.74 × 100 million RMB), whereas DMU 7 has to increase its interest expenses to 971.27 × 100 million RMB to achieve its target outputs. In addition, DMUs 2, 3 and 6 only change their interest expenses without considering the two other inputs.

Fourth, the production target and net percentage increase of intermediate measure are presented in the fifth and last columns of Table 5. We can find that some intermediate measures of DMUs increase while others decrease. This is because we do not have to force the intermediate measures of DMUs to increase. DMUs 1, 2, 8, 12, 14, and 15 should reduce their deposits, while the other DMUs should strive to get more deposits. DMUs 16 should increase their intermediate measure by 15%, and the reduction ratio of DMU 14 reaches up to 17.66%. Bank managers may always seek for more deposits. However, redundant deposits improve the stage 1 efficiency yet reduce the stage 2 efficiency. In this case, when the overall efficiency of the bank is assumed to stay the same, reducing the number of deposits becomes a viable option.

#### Scenario 2

The plans for increasing the desirable outputs by 15% and undesirable outputs by 5% is shown in Table 6. From the input–output perspective, with the same efficiency, having less outputs corresponds to having less inputs. Therefore, the new plan of inputs in this scenario is less than that in scenario 1. Specifically, the resource plans of DMUs 14, 15, and 16 are larger than those obtained in scenario 1, thereby suggesting that if these three DMUs want to minimize the increase in their undesirable outputs, they need to shoulder more costs compared with the other DMUs. Therefore, these DMUs must improve their technologies to save costs. Excluding two DMUs with NF, 8 DMUs can reduce their intermediate measure to a percentage greater than that recorded in scenario 1. Meanwhile, for DMUs 1 to 13 (excluding DMUs 9 and 10), the growth ratio of their intermediate measure is lower in scenario 2 than in scenario 1.

**Table 6:** Plans of inputs and intermediate measure for increasing the desirable outputs by 15% and undesirable outputs by 5%.

DMU	Plans of inputs and intermediate measure				Net percentage increase (%)			
	α_1_	α_2_	α_3_	γ	P_1_	P_2_	P_3_	P_4_
1	2007.06	3505.64	441902.00	137286.57	13.50	8.27	0.00	-6.10
2	1693.97	2606.88	473766.00	113890.15	0.00	9.91	0.00	-3.58
3	1478.42	2698.68	251617.00	109603.36	0.00	14.64	0.00	8.54
4	1674.47	2926.35	368410.00	120847.32	7.49	13.99	0.00	-1.13
5	629.93	1334.90	99919.00	45058.73	17.06	3.77	0.00	8.37
6	458.96	835.80	51667.00	28474.36	0.00	12.06	0.00	2.60
7	329.08	916.18	38803.00	26739.55	0.19	17.99	0.00	0.84
8	330.66	944.93	38976.00	21202.96	24.29	2.01	0.00	-12.37
9	NF	NF	NF	NF	NF	NF	NF	NF
10	NF	NF	NF	NF	NF	NF	NF	NF
11	240.15	571.21	28369.00	12075.24	12.86	8.98	0.00	-0.78
12	184.45	416.36	25043.00	10645.38	4.66	11.47	0.00	-9.60
13	262.68	692.20	31464.00	15791.08	26.40	0.00	0.00	-1.63
14	94.33	380.11	11059.41	7118.66	20.30	20.30	20.30	-14.69
15	38.18	153.85	4476.33	2630.99	17.29	31.81	2.74	1.13
16	53.58	163.74	6310.00	2908.17	20.40	33.81	0.00	24.31

Note: NF means No feasible solution.

#### Summary

By comparing the two scenarios, we can get the following conclusions. ① Relative to the third input (labor), the first two inputs (operation cost and interest expense) are more likely to be changed, and the intermediate measure (deposits) may increase and may decrease. ② For some DMUs, the plan is not implemented, such as DMU 9 and 10. ③ In the case of increasing the same proportion of desirable output, a lower proportion of undesirable output does not necessarily mean less investment, but may require more cost input to achieve the output target, such as DMU 13, 14 and 15.

### Suggestions for banks

Increasing the non-performing loans and various incomes by the same proportion is a relatively easy goal to achieve in the short term. Achieving such goal requires not only increasing the costs but also increasing the number of employees.

The amount of deposits does not need to be maximized. To ensure that the same efficiency will be achieved, excessive deposits may be used as cash reserves because they cannot be used to generate profits in a timely manner. Although having more deposits is one of the output targets set in stage 1, these deposits can significantly increase the interest costs and subsequently reduce the efficiency of banks in stage 1. At the same time, excessive deposits can also lead to poor efficiency in stage 2.

With the goal of generating revenue while minimizing their non-performing loans, some banks have to pay more to reach their objectives, while others may be completely unable to reach their goals. For the former, banks must improve the business level of their employees, expand the role of input costs, and efficiently utilize their adsorbed deposits. For the latter, these banks should focus on the credit quality of their borrowers, minimize their non-performing loans, and actively recover these loans to reduce their level.

## Conclusions

This paper formulates some resource plans for banks whose outputs are expected to increase in the short term. The overall efficiency of 16 Chinese listed commercial banks with a two-stage system must be kept unchanged. Two-stage inverse DEA models are developed in this work to solve such problem. These models consider the weak disposability of undesirable outputs [55]. Three scenarios are also designed to formulate resource plans for banks. Given their weak disposability, some DMUs have no feasible investment plan to complete the target of outputs in some cases. Meanwhile, the inputs of other DMUs should be increased in different degrees. Most DMUs do not increase their third type of input, namely, labor (*x*_*3*_). In the short term, banks cannot possibly increase their number of employees. Intermediate measures (deposits), as the regulator of these two stages, have no obvious law of change, that is, some DMUs’ intermediate measures increase while those of other DMUs decrease. Therefore, banks should 1) increase their operation costs and interest expenses to complete their short-term tasks, 2) control their amount of deposits at a reasonably efficient level, 3) improve their employees’ business levels, and 4) critically review the creditworthiness of their borrowers. The models proposed in this paper are not only suitable for banks but can also be applied to other two-stage systems, such as supply chain systems.

This study can be extended to several directions. For instance, our approaches can be applied to DMUs with variable returns to scale. The structure of DMUs can also be extended to highly complex networks [56]. However, this paper ignores the efficiency of sub-stages, and future studies may consider keeping both the overall and sub-stage efficiencies invariable.

## Appendix

**Proof for Theorem 1**: The production possibility set (PPS) of model (5) can be expressed as

$$T=\{\;\left(x,y,u,z\right)\;|\begin{array}{l} \;x\geq \sum_{j=1}^{n}\lambda_{j}x_{ij}\\ \;z\leq \sum_{j=1}^{n}\lambda_{j}z_{dj}\\ \;z\geq \sum_{j=1}^{n}\pi_{j}z_{dj}\\ \;y\leq \sum_{j=1}^{n}\pi_{j}y_{rj}\\ \;u=\sum_{j=1}^{n}\pi_{j}u_{kj}\\ \;\lambda_{j},\pi_{j}\geq 0,j=1,2,\ldots ,n \end{array}\;\}.$$ (9)

When the weak Pareto optimal solution of model (7) is ($\alpha_{i0}^{*},\lambda_{j}^{*},\pi_{j}^{*},\gamma_{d0}^{*}$), we have

$$\sum_{j=1}^{n}\lambda_{j}^{*}x_{ij}\leq \theta_{0}^{*}\alpha_{i0}^{*},\;i=1,2,\ldots ,m$$ (10)

$$\sum_{j=1}^{n}\lambda_{j}^{*}z_{dj}\geq \gamma_{d0}^{*}\;\;\;\;\;d=1,2,\ldots ,t$$ (11)

$$\sum_{j=1}^{n}\pi_{j}^{*}z_{dj}\leq \gamma_{d0}^{*},\;\;\;d=1,2,\ldots ,t$$ (12)

$$\sum_{j=1}^{n}\pi_{j}^{*}y_{rj}\geq \beta_{r0},\;\;\;r=1,2,\ldots ,s$$ (13)

$$\sum_{j=1}^{n}\pi_{j}^{*}u_{kj}=\mu_{k0},\;\;\;k=1,2,\ldots ,h$$ (14)

which suggest that $\left(\alpha_{i0}^{*},\gamma_{d0}^{*},\beta_{r0}^{*},\mu_{r0}^{*}\right)\;=\;\left(x_{i0}+\Delta x_{i0},\;z_{d0}\pm \Delta z_{d0},\;y_{r0}+\Delta y_{r0},\;u_{k0}+\Delta u_{k0}\right)\in T$. Here, we explain why γ* = *z*_*d*0_ ± Δ*z*_*d*0_. Given that the two constraints (11) and (12) limit the intermediate measures and that these two limits are reversed, the intermediate measures may both increase and decrease.

We use model (8) to evaluate the efficiency of DMU_*n*+1_ whose inputs, intermediate measures, and outputs are changed. Therefore, the PPS based on model (8) can be derived as

$$T_{n+1}=\{\;\left(x,y,u,z\right)\;|\begin{array}{l} \;x\geq \sum_{j=1}^{n}\lambda_{j}x_{ij}+\lambda_{n+1}\alpha_{i0}^{*}\\ \;\gamma \leq \sum_{j=1}^{n}\lambda_{j}z_{dj}+\lambda_{n+1}\gamma_{d0}^{*}\\ \;\gamma \geq \sum_{j=1}^{n}\lambda_{j}z_{dj}+\lambda_{n+1}\gamma_{d0}^{*}\\ \;y\leq \sum_{j=1}^{n}\pi_{j}y_{rj}+\pi_{n+1}\beta_{r0}\\ \;u=\sum_{j=1}^{n}\pi_{j}u_{kj}+\pi_{n+1}\mu_{k0}\\ \;\lambda_{j},\pi_{j}\geq 0,j=1,2,\ldots ,n+1 \end{array}\;\}.$$ (15)

PPS *T*_*n*+1_ is the same as PPS *T* and consists of PPS *T* and DMU_*n*+1_. The difference between PPS *T*_*n*+1_ and PPS *T* is only DMU_*n*+1_, that is, (*x*_*i*0_ + Δ*x*_*i*0_, *z*_*d*0_ ± Δ*z*_*d*0_, *y*_*r*0_ + Δ*y*_*r*0_, *u*_*k*0_ + Δ*u*_*k*0_).

We know that (*x*_*i*0_ + Δ*x*_*i*0_, *z*_*d*0_ ± Δ*z*_*d*0_, *y*_*r*0_ + Δ*y*_*r*0_, *u*_*k*0_ + Δ*u*_*k*0_) ∈ *T*, so DMU_*n*+1_ does not change the efficient frontier of PPS *T*_*n*+1_ compared with PPS *T*. Therefore, the PPS *T*_*n*+1_ based on model (7) can be replaced by PPS *T*. Model (8) can then be rewritten as follows:

$$\begin{array}{l} \text{min}\theta_{0}\\ \text{s}.\text{t}.\\ \sum_{j=1}^{n}\lambda_{j}x_{ij}\leq \theta_{0}\alpha_{i0},\;\;\;i=1,2,\ldots ,m\\ \sum_{j=1}^{n}\lambda_{j}z_{dj}\geq \gamma_{d0},\;d=1,2,\ldots ,t\\ \sum_{j=1}^{n}\pi_{j}z_{dj}\leq \gamma_{d0},\;d=1,2,\ldots ,t\\ \sum_{j=1}^{n}\pi_{j}y_{rj}\geq \beta_{r0},\;r=1,2,\ldots ,s\\ \sum_{j=1}^{n}\pi_{j}u_{kj}=\mu_{k0},\;k=1,2,\ldots ,s\\ \lambda_{j}\geq 0,\;j=1,2,\ldots ,n\text{+}1\\ \pi_{j}\geq 0,\;j=1,2,\ldots ,n\text{+}1 \end{array}$$ (16)

Suppose that the optimal value of model (12) is $\theta_{0}^{+}$. Given that the constraints set (9) satisfies model (16), we have $\theta_{0}^{+}\leq \theta_{0}^{*}$. If we can prove that $\theta_{0}^{+}\neq \theta_{0}^{*}$ is invalid, then $\theta_{0}^{+}\;=\;\theta_{0}^{*}$ is valid. Assume $\theta_{0}^{+}<\theta_{0}^{*}$, then $\theta_{0}^{+}\;=\;k\theta_{0}^{*}$, where 0 < *k* < 1. We have

$$\begin{array}{l} \sum_{j=1}^{n}\lambda_{j}x_{ij}\leq \theta_{0}^{+}\alpha =k\theta_{0}^{*}\alpha ,\;i=1,2,\ldots ,m\\ \sum_{j=1}^{n}\lambda_{j}z_{dj}\geq \gamma_{d0},\;d=1,2,\ldots ,t\\ \sum_{j=1}^{n}\pi_{j}z_{dj}\leq \gamma_{d0},\;d=1,2,\ldots ,t\\ \sum_{j=1}^{n}\pi_{j}y_{rj}\geq \beta_{r0},\;r=1,2,\ldots ,s\\ \sum_{j=1}^{n}\pi_{j}u_{kj}=\mu_{k0},\;k=1,2,\ldots ,h\\ \lambda_{j}\geq 0,\;j=1,2,\ldots ,n\text{+}1\\ \pi_{j}\geq 0,\;j=1,2,\ldots ,n\text{+}1\\ \gamma_{d0}\geq 0,\;d=1,2,\ldots ,t \end{array}$$ (17)

which means that ($k\alpha_{i0}^{*},\lambda_{j}^{*},\pi_{j}^{*},\gamma_{d0}^{*}$) is a feasible solution for model (7). However, this result contradicts the assumption that the weak Pareto optimal solution of model (7) is ($\alpha_{i0}^{*},\lambda_{j}^{*},\pi_{j}^{*},\gamma_{d0}^{*}$) in the beginning of the proof because $k\alpha_{i0}^{*}\leq \alpha_{i0}^{*}$. Therefore, the assumption $\theta_{0}^{+}<\theta_{0}^{*}$ is invalid, and $\theta_{0}^{+}\;=\;\theta_{0}^{*}$.

## Supporting information

S1 File Table A Raw Data of 16 Chinese listed commercial banks for 2013. Table B Relevant data in manuscript at increasing both desirable and undesirable outputs by same percentage. Table C Relevant data in manuscript at increasing desirable and undesirable outputs by different percentage. Table D Relevant data in manuscript at increasing desirable outputs and keeping undesirable outputs unchanged.
(XLSX)

## References

[1] Wang K, Huang W, Wu J, Liu YN. Efficiency measures of the Chinese commercial banking system using an additive two-stage DEA. Omega, 2014, 44: 5–20. 10.1016/j.omega.2013.09.005

[2] Matthews K. Risk management and managerial efficiency in Chinese banks: A network DEA framework. Omega, 2013, 41(2): 207–215. 10.1016/j.omega.2012.06.003

[3] Shaban M, James GA. The effects of ownership change on bank performance and risk exposure: Evidence from Indonesia. Journal of Banking & Finance, 2018, 88: 483–497. 10.1016/j.jbankfin.2017.02.002

[4] Kao C, Liu ST. Predicting bank performance with financial forecasts: A case of Taiwan commercial banks. Journal of Banking & Finance, 2004, 28(10): 2353–2368. 10.1016/j.jbankfin.2003.09.008

[5] Charnes A, Cooper WW, Rhodes E. Measuring the efficiency of decision making units. European journal of operational research, 1978, 2(6): 429–444. 10.1016/0377-2217(78)90138-8

[6] An QX, Wang ZR, Emrouznejad A, Zhu QY, Chen XH. Efficiency evaluation of parallel interdependent processes systems: an application to Chinese 985 Project universities. International Journal of Production Research, 2018: 1–1310.

[7] Wu J, Zhu QY, Chu JF, An QX, Liang L. A DEA-based approach for allocation of emission reduction tasks. International Journal of Production Research, 2016, 54(18): 5618–5633. 10.1080/00207543.2016.1194537

[8] Dong G, Wang ZZ, Mao XQ. Production efficiency and GHG emissions reduction potential evaluation in the crop production system based on emergy synthesis and nonseparable undesirable output DEA: A case study in Zhejiang Province, China. PloS one, 2018, 13(11): e0206680 10.1371/journal.pone.0206680 30383842 PMC6211734

[9] Lozano S, Villa G, Eguía I. Data envelopment analysis with multiple modes of functioning. Application to reconfigurable manufacturing systems. International Journal of Production Research, 2017, 55(24): 7566–7583. 10.1080/00207543.2017.1391418

[10] Li F, Song J, Dolgui A, Liang L. Using common weights and efficiency invariance principles for resource allocation and target setting. International Journal of Production Research, 2017, 55(17): 4982–4997. 10.1080/00207543.2017.1287450

[11] Flokou A, Aletras V, Niakas D. A window-DEA based efficiency evaluation of the public hospital sector in Greece during the 5-year economic crisis. PloS one, 2017, 12(5): e0177946 10.1371/journal.pone.0177946 28542362 PMC5441611

[12] Zheng WH, Sun H, Zhang PL, Zhou GJ, Jin QY, Lu XQ. A four-stage DEA-based efficiency evaluation of public hospitals in China after the implementation of new medical reforms. PloS one, 2018, 13(10): e0203780 10.1371/journal.pone.0203780 30281620 PMC6169866

[13] Wei QL, Zhang JZ, Zhang XS. An inverse DEA model for inputs/outputs estimate. European Journal of Operational Research, 2000, 121(1): 151–163. 10.1016/S0377-2217(99)00007-7

[14] Hassanzadeh A, Yousefi S, Saen R, Hosseininia S. How to assess sustainability of countries via inverse data envelopment analysis?. Clean Technologies and Environmental Policy, 2018, 20(1): 29–40.

[15] Chen L, Wang YM, Lai FJ, Feng F. An investment analysis for China’s sustainable development based on inverse data envelopment analysis. Journal of cleaner production, 2017, 142: 1638–1649. 10.1016/j.jclepro.2016.11.129

[16] Lim DJ. Inverse DEA with frontier changes for new product target setting. European Journal of Operational Research, 2016, 254(2): 510–516. 10.1016/j.ejor.2016.03.059

[17] Hadi-Vencheh A, Hatami-Marbini A, Beigi ZG, Gholami K. An inverse optimization model for imprecise data envelopment analysis. Optimization, 2015, 64(11): 2441–2454. 10.1080/02331934.2014.974599

[18] Gattoufi S, Amin GR, Emrouznejad A. A new inverse DEA method for merging banks. IMA Journal of Management Mathematics, 2014, 25(1): 73–87. 10.1093/imaman/dps027

[19] Frija A, Wossink A, Buysse J, Speelman S, Van Huylenbroeck G. Irrigation pricing policies and its impact on agricultural inputs demand in Tunisia: A DEA-based methodology. Journal of environmental management, 2011, 92(9): 2109–2118. 10.1016/j.jenvman.2011.03.013 21524839

[20] Hadi-Vencheh A, Foroughi AA, Soleimani-damaneh M. A DEA model for resource allocation. Economic Modelling, 2008, 25(5): 983–993. 10.1016/j.econmod.2008.01.003

[21] Jahanshahloo GR, Vencheh AH, Foroughi AA, Matin RK. Inputs/outputs estimation in DEA when some factors are undesirable. Applied Mathematics and Computation, 2004, 156(1): 19–32. 10.1016/S0096-3003(03)00814-2

[22] Yan H, Wei QL, Hao G. DEA models for resource reallocation and production input/output estimation. European Journal of Operational Research, 2002, 136(1): 19–31. 10.1016/S0377-2217(01)00046-7

[23] Chen XG, Skully M, Brown K. Banking efficiency in China: Application of DEA to pre-and post-deregulation eras: 1993–2000. China Economic Review, 2005, 16(3): 229–245. 10.1016/j.chieco.2005.02.001

[24] Yao SJ, Han ZW, Feng GF. Ownership reform, foreign competition and efficiency of Chinese commercial banks: A non-parametric approach. World Economy, 2008, 31(10): 1310–1326. 10.1111/j.1467-9701.2008.01130.x

[25] Ariff M, Luc C. Cost and profit efficiency of Chinese banks: A non-parametric analysis. China Economic Review, 2008, 19(2): 260–273. 10.1016/j.chieco.2007.04.001

[26] Boussemart JP, Leleu H, Shen ZY, Vardanyan M, Zhu N. Decomposing banking performance into economic and credit risk efficiencies. European Journal of Operational Research, 2019, 277(2): 719–726. 10.1016/j.ejor.2019.03.006

[27] Izadikhah M, Tavana M, Di Caprio D, Santos-Arteaga FJ. A novel two-stage DEA production model with freely distributed initial inputs and shared intermediate outputs. Expert Systems with Applications, 2018, 99, 213–230. 10.1016/j.eswa.2017.11.005

[28] An QX, Meng FY, Ang S, Chen XH. A new approach for fair efficiency decomposition in two-stage structure system. Operational Research, 2018, 18(1), 257–272. 10.1007/s12351-016-0262-9

[29] Fukuyama H, Matousek R. Modelling bank performance: A network DEA approach. European Journal of Operational Research, 2017, 259(2), 721–732. 10.1016/j.ejor.2016.10.044

[30] Ding JJ, Dong W, Liang L, Zhu J. Goal congruence analysis in multi-Division Organizations with shared resources based on data envelopment analysis. European Journal of Operational Research, 2017, 263(3), 961–973. 10.1016/j.ejor.2017.06.040

[31] Wanke P, Maredza A, Gupta R. Merger and acquisitions in South African banking: A network DEA model. Research in International Business and Finance, 2017, 41, 362–376. 10.1016/j.ribaf.2017.04.055

[32] Degl’Innocenti M, Kourtzidis SA, Sevic Z, Tzeremes NG. Bank productivity growth and convergence in the European Union during the financial crisis. Journal of Banking & Finance, 2017, 75, 184–199. 10.1016/j.jbankfin.2016.11.016

[33] Hadi-Vencheh A, Foroughi AA. A generalized DEA model for inputs/outputs estimation. Mathematical and Computer Modelling, 2006, 43(5–6): 447–457. 10.1016/j.mcm.2005.08.005

[34] Zhang M, Cui JC. The extension and integration of the inverse DEA method. Journal of the Operational Research Society, 2016, 67(9): 1212–1220. 10.1057/jors.2016.2

[35] Jahanshahloo GR, Lotfi FH, Shoja N, Tohidi G, Razavyan S. Sensitivity of efficiency classifications in the inverse DEA models. Applied Mathematics and computation, 2005, 169(2): 905–916. 10.1016/j.amc.2004.09.093

[36] Ghiyasi M. On inverse DEA model: The case of variable returns to scale. Computers & Industrial Engineering, 2015, 87: 407–409. 10.1016/j.cie.2015.05.018

[37] Lim S, Zhu J. A note on two-stage network DEA model: Frontier projection and duality. European Journal of Operational Research, 2016, 248(1): 342–346. 10.1016/j.ejor.2015.06.050

[38] Ghiyasi M. Inverse DEA based on cost and revenue efficiency. Computers & Industrial Engineering, 2017, 114: 258–263. 10.1016/j.cie.2017.10.024

[39] Lin HT. An efficiency-driven approach for setting revenue target. Decision Support Systems, 2010, 49(3): 311–317. 10.1016/j.dss.2010.03.006

[40] Amin GR, Emrouznejad A, Gattoufi S. Modelling generalized firms’ restructuring using inverse DEA. Journal of Productivity Analysis, 2017, 48(1): 51–61. 10.1007/s11123-017-0501-y

[41] Ghiyasi M. Industrial sector environmental planning and energy efficiency of Iranian provinces. Journal of cleaner production, 2017, 142: 2328–2339. 10.1016/j.jclepro.2016.11.044

[42] Amin GR, Al-Muharrami S. A new inverse data envelopment analysis model for mergers with negative data. IMA Journal of Management Mathematics, 2016, 29(2): 137–149. 10.1093/imaman/dpw016

[43] Matin RK, Amin GR, Emrouznejad A. A modified semi-oriented radial measure for target setting with negative data. Measurement, 2014, 54: 152–158. 10.1016/j.measurement.2014.04.018

[44] Modhej D, Sanei M, Shoja N, HosseinzadehLotfi F. Integrating inverse data envelopment analysis and neural network to preserve relative efficiency values. Journal of Intelligent & Fuzzy Systems, 2017, 32(6): 4047–4058. 10.3233/JIFS-152271

[45] Seiford LM, Zhu J. Profitability and marketability of the top 55 US commercial banks. Management science, 1999, 45(9): 1270–1288. 10.1287/mnsc.45.9.1270

[46] Lertworasirikul S, Charnsethikul P, Fang SC. Inverse data envelopment analysis model to preserve relative efficiency values: The case of variable returns to scale. Computers & Industrial Engineering, 2011, 61(4): 1017–1023. 10.1016/j.cie.2011.06.014

[47] Chen Y, Cook WD, Zhu J. Deriving the DEA frontier for two-stage processes. European Journal of Operational Research, 2010, 202(1): 138–142. 10.1016/j.ejor.2009.05.012

[48] Liu WB, Zhou ZB, Ma CQ, Liu DB, Shen WF. Two-stage DEA models with undesirable input-intermediate-outputs. Omega, 2015, 56: 74–87. 10.1016/j.omega.2015.03.009

[49] Färe R, Grosskopf S. Modeling undesirable factors in efficiency evaluation: comment. European Journal of Operational Research, 2004, 157(1): 242–245. 10.1016/S0377-2217(03)00191-7

[50] Paradi JC, Zhu HY. A survey on bank branch efficiency and performance research with data envelopment analysis. Omega, 2013, 41(1): 61–79. 10.1016/j.omega.2011.08.010

[51] Fukuyama H, Weber WL. A slacks-based inefficiency measure for a two-stage system with bad outputs. Omega, 2010, 38(5): 398–409. 10.1016/j.omega.2009.10.006

[52] Paradi JC, Rouatt S, Zhu HY. Two-stage evaluation of bank branch efficiency using data envelopment analysis. Omega, 2011, 39(1): 99–109. 10.1016/j.omega.2010.04.002

[53] Zha Y, Liang NN, Wu MG, Bian YW. Efficiency evaluation of banks in China: A dynamic two-stage slacks-based measure approach. Omega, 2016, 60: 60–72. 10.1016/j.omega.2014.12.008

[54] An QX, Chen HX, Wu J, Liang L. Measuring slacks-based efficiency for commercial banks in China by using a two-stage DEA model with undesirable output. Annals of Operations Research, 2015, 235(1): 13–35. 10.1007/s10479-015-1987-1

[55] Kuosmanen T. Weak disposability in nonparametric production analysis with undesirable outputs. American Journal of Agricultural Economics, 2005, 87(4): 1077–1082. 10.1111/j.1467-8276.2005.00788.x

[56] Vanhoucke M. Planning projects with scarce resources: Yesterday, today and tomorrow’s research challenges. Frontiers of Engineering Management, 2018, 5(2): 133–149. 10.15302/J-FEM-2018088

